# The Role of Nuclear Receptors in Prostate Cancer

**DOI:** 10.3390/cells8060602

**Published:** 2019-06-17

**Authors:** Masaki Shiota, Naohiro Fujimoto, Eiji Kashiwagi, Masatoshi Eto

**Affiliations:** 1 Department of Urology, Graduate School of Medical Sciences, Kyushu University, Fukuoka 812-8582, Japan; kashiwag@uro.med.kyushu-u.ac.jp (E.K.); etom@uro.med.kyushu-u.ac.jp (M.E.); 2 Department of Urology, School of Medicine, University of Occupational and Environmental Health, Kitakyushu 807-8555, Japan; n-fuji@med.uoeh-u.ac.jp

**Keywords:** androgen receptor, estrogen receptor, glucocorticoid receptor, mineralocorticoid receptor, nuclear receptor, progesterone receptor, prostate cancer, steroid receptor

## Abstract

The nuclear receptor (NR) superfamily consists of 48 members that are divided into seven subfamilies. NRs are transcription factors that play an important role in a number of biological processes. The NR superfamily includes androgen receptor, which is a key player in prostate cancer pathogenesis, suggesting the functional roles of other NRs in prostate cancer. The findings on the roles of NRs in prostate cancer thus far have shown that several NRs such as vitamin D receptor, estrogen receptor β, and mineralocorticoid receptor play antioncogenic roles, while other NRs such as peroxisome proliferator-activated receptor γ and estrogen receptor α as well as androgen receptor play oncogenic roles. However, the roles of other NRs in prostate cancer remain controversial or uninvestigated. Further research on the role of NRs in prostate cancer is required and may lead to the development of novel preventions and therapeutics for prostate cancer.

## 1. Introduction

Prostate cancer is primarily characterized by a dependence on the axis of androgen and its cognitive receptor, the nuclear receptor (NR) androgen receptor (AR), which plays roles in carcinogenesis, cancer development, disease progression, and treatment resistance [1]. Therefore, AR axis-targeting therapeutics such as androgen-deprivation therapy and antiandrogens have been the gold-standard treatments for recurrent or advanced prostate cancer [2].

The NR superfamily consists of 48 members that are divided into seven subfamilies [3]. The classification into subfamilies was determined by phylogenetic tree analysis based on their sequences [4]. NRs are transcription factors that play important functions in various biological processes including growth, development, metabolism, reproduction, and inflammation [3]. Except for subfamily 0, the structure of NRs is composed of five or six domains: A–E(F) (A/B, N-terminal domain; C, DNA-binding domain; D, hinge region; E/(F), ligand-binding domain [LBD]). Most NRs are regulated by endogenous small lipophilic ligands, such as steroids, retinoids, and phospholipids, while some NRs are still classified as orphan receptors with no identified ligand thus far [3]. Ligand binding induces conformational changes within the NR, leading to its translocation into the nucleus and binding to cognitive binding sites on DNA throughout the genome [3]. Coregulator proteins, chromatin remodeling factors, and the general transcriptional machinery are then recruited to regulate the expression of NR target genes [3]. Notably, multiple NRs are involved in various aspects of reproduction and several NRs also function in the prostate. In addition, NR-regulated biological processes, such as growth, development, metabolism, and inflammation, are critical factors for prostate cancer pathogenesis [5,6]. Accordingly, other NRs have been speculated to function in prostate cancer pathogenesis in addition to their roles in physiological and pathological conditions. Most NRs are expressed and functional in the nonmalignant prostate as well as during prostate cancer or are dysregulated in prostate cancer [7,8]. Multiple NRs, including retinoic acid receptors (RARs), retinoid X receptors (RXRs), vitamin D receptor (VDR), liver X receptors (LXRs), peroxisome proliferator-activated receptors (PPARs), farnesoid X receptors (FXRs), and chicken ovalbumin upstream promoter transcription factor γ (COUP-TFγ), are strongly expressed in nonmalignant prostate epithelial RWPE-1 cells and normal human prostate epithelial cells [7]. In contrast, the expression levels of several NRs including LXRα, LXRβ, RARγ, and RXRα are downregulated in malignant-transformed prostate epithelial RWPE-2 cells as well as clinical prostate cancer samples [7]. These studies suggest that better clarification of the precise roles of NRs in prostate cancer may not only help better elucidate their cellular functions but may also lead to the development of novel prevention and therapeutic strategies for prostate cancer.

In this review, we summarize the roles of NRs in prostate cancer according to the classification into subfamilies with a focus on NRs other than AR (Table 1).

## 2. Subfamilies of NRs

### 2.1. Subfamily 0

This subfamily includes the atypical NRs, including dosage-sensitive sex reversal-adrenal hypoplasia congenital critical region on the X chromosome, Gene 1 (DAX1) and small heterodimer partner (SHP) [3]. These two NRs are unique in that their structures contain only a LBD, which interacts with the LBDs of other NRs to regulate the transcriptional activity of the interacting NRs [3].

#### 2.1.1. Dosage-Sensitive Sex Reversal-Adrenal Hypoplasia Congenital Critical Region on the X Chromosome, Gene 1 (DAX1)

DAX1 is predominantly expressed in male and female reproductive organs, including testis, prostate, ovary, and adrenal gland, where DAX1 regulates steroidogenesis, development, and reproduction via interactions with other NRs [9]. The expression pattern of DAX1 in nonmalignant prostate and prostate cancer is controversial. Strong nuclear expression of DAX1 was observed in normal prostatic epithelial cells, but reduced expression was observed in benign prostatic hyperplasia (BPH) and androgen-independent PC-3 prostate cancer cells [9,10,11]. However, DAX1 exhibited a variable expression pattern in nuclei of prostate cancer cells and its expression was inversely correlated with Gleason score [12]. Functionally, DAX1 repressed AR activity in androgen-dependent LNCaP prostate cancer cells [9]. However, a biological role for DAX1 in prostate cancer has not been reported so far.

#### 2.1.2. Short Heterodimeric Partner (SHP)

SHP is abundantly expressed mainly in the enterohepatic system, including in liver and intestine, where SHP regulates bile acid synthesis and cholesterol homeostasis [13]. A recent study showed downregulation of SHP in several human prostate cancer cell lines compared with nonmalignant prostatic epithelial cells [14]. SHP expression was also reduced in prostate cancer tissues compared with BPH tissues [15]. Moreover, decreased SHP expression in prostate cancer tissues was associated with increased risks of recurrence and progression after radical prostatectomy [16]. Similar to DAX1, SHP was shown to repress AR transcriptional activity in LNCaP cells [14]. Furthermore, synthetic SHP agonists could induce apoptosis and suppress the in vitro growth of androgen-independent DU-145 prostate cancer cells [17]. SHP overexpression also suppressed cell proliferation in prostate cancer cells [16], increased apoptosis in LNCaP cells [18], and induced cell cycle arrest at G1 phase in PC-3 cells [19], suggesting SHP exerts an antioncogenic role in prostate cancer.

**Figure 1:**
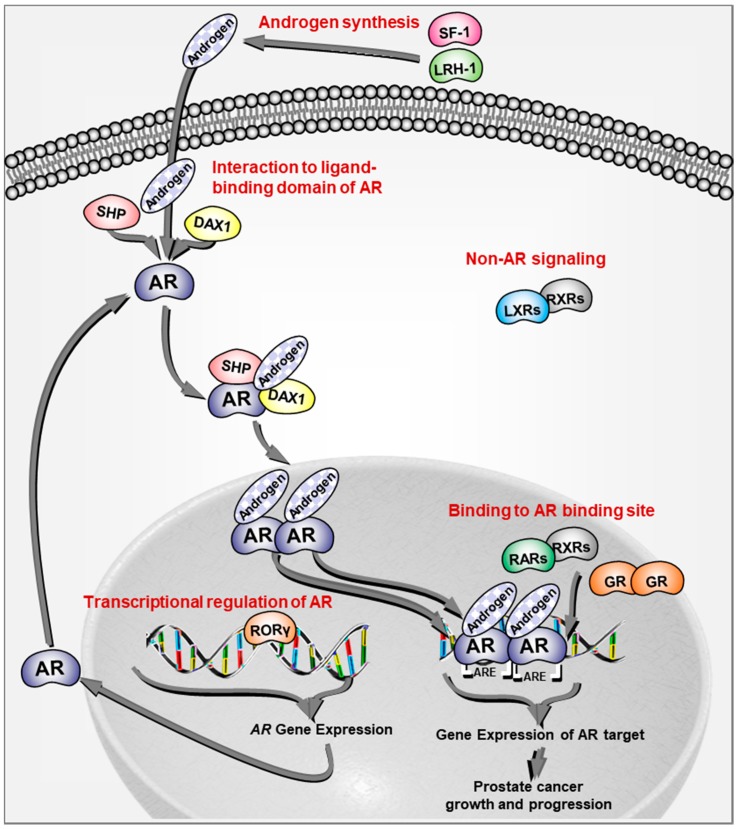
Schematic representation of the signaling pathways on androgen receptor signaling in prostate cancer by nuclear receptors. Nuclear receptors regulate androgen receptor (AR) signaling via various mechanisms including androgen synthesis, transcriptional regulation of AR, interaction with the ligand-binding domain of AR, and binding to AR-binding sites in addition to non-AR signaling.

Taken together, these studies indicate that the NRs in subfamily 0 play an antioncogenic role in prostate cancer partially through an inhibitory effect on AR, which may be mediated by their interactions with the LBD of AR (Figure 1).

### 2.2. Subfamily 1

This large subfamily consists of thyroid hormone receptors, RARs, PPARs, reverse-Erb receptors (REV-ERBs), retinoic acid receptor-related orphan receptors (RORs), FXRs, LXRs, and VDR [3]. These receptors are regulated by a variety of lipophilic molecules such as thyroid hormone, fatty acids, bile acids, and sterols [3].

#### 2.2.1. Retinoic Acid Receptors (RARs)

Retinoic acid, the major bioactive metabolite of retinol or vitamin A, induces pleiotropic effects on cell growth and differentiation in various organs [20]. Retinoic acid activity is mediated primarily by members of the RAR subfamily (RARα, RARβ, and RARγ), which form heterodimers with members of the RXR subfamily and regulate the expression of target genes through binding to retinoic acid-response elements [20]. Data from Memorial Sloan Kettering Cancer Center [21] and TCGA Prostate Adenocarcinoma showed that RARγ was significantly and uniquely downregulated in prostate cancer compared with other cancers [22]. In addition, the RARβ promoter methylation status was higher in prostate cancer compared with nonmalignant tissues, suggesting reduced expression of RARβ in prostate cancer [23]. In LNCaP cells, RARγ signaling was shown to suppress AR signaling by competitive binding to AR-binding sites (Figure 1) [24,25]. Consistent with these findings, agonists for RARs alone and in combination with other agents suppressed oncogenic characteristics in prostate cancer cells, including cell proliferation, and reduced prostate tumor growth [24,26,27,28,29]. Interestingly, a selective agonist for RARγ, but not a selective agonist for RARα, showed a suppressive effect on cell proliferation in DU-145 cells, which was reversed by RAR antagonist treatment [30]. In addition, RARβ overexpression was shown to reduce prostate cancer cell proliferation [31]. Taken together, RARβ and RARγ seem to play an antioncogenic role in prostate cancer through an inhibitory effect on AR signaling by competitive binding to AR-binding sites (Figure 1).

#### 2.2.2. Peroxisome Proliferator-Activated Receptors (PPARs)

The PPAR subfamily members (PPARα, PPARβ/δ, and PPARγ) are fatty acid-activated transcription factors that are involved in several physiological processes including modulation of cellular differentiation, development, and metabolism [32]. Upon ligand binding of fatty acids to PPARs, these NRs translocate into the nucleus as heterodimers with RXRs and bind to peroxisome proliferator response elements in target genes to regulate target gene expression [33]. PPARα and PPARβ/δ expressions are detected among normal prostate, BPH, and prostate cancer tissues, while expression of PPARγ was observed in prostate cancer but not in normal prostate and BPH [34]. Furthermore, PPARγ expression was shown to increase with cancer grade/stage and correlate with poor survival in prostate cancer patients, suggesting that PPARγ plays an oncogenic role in prostate cancer development and progression [35,36,37]. Functionally, the effect on AR signaling by PPARγ is controversial and dependent on the cellular context [38]. PPARγ was originally thought to exert antioncogenic properties in prostate cancer because PPARγ agonists inhibited the growth of prostate cancer cells. However, additional studies found that PPARγ agonists inhibited cell growth independent of PPARγ [37], and fatty acids promoted tumorigenesis [39]. Indeed, recent studies showed that PPARγ activation promoted prostate cancer progression [40,41], suggesting that PPARγ inhibition might be useful in prevention and treatment for prostate cancer [42]. Intriguingly, a previous study reported that AR signaling negatively regulated PPARγ signaling, suggesting that PPARγ function may be augmented in castrated conditions [43]. The role of PPARβ/δ in prostate cancer is still controversial [44,45,46], indicating complex functions of PPARs in prostate cancer biology.

#### 2.2.3. Retinoic Acid Receptor-Related Orphan Receptors (RORs)

The ROR superfamily proteins (RORα, RORβ, and RORγ) are generally classified as orphan receptors, but sterols have been suggested as possible ligands. RORs regulate gene expression by binding to ROR response elements as monomers [3,47,48]. RORγ expression is upregulated in prostate cancer and further increased in castration-resistant prostate cancer (CRPC). RORγ drives AR expression while selective RORγ antagonists inhibit AR expression and prostate tumor growth (Figure 1) [49,50], suggesting an oncogenic role for RORγ and its potential as a therapeutic target in prostate cancer. In contrast, an antioncogenic role for RORα in prostate cancer was indicated by its tumor suppression and anti-invasion functions [51,52]. Taken together, these findings suggest distinct functions of RORs in prostate cancer.

#### 2.2.4. Farnesoid X Receptors (FXRs)

The FXR subfamily (FXRα and FXRβ, pseudogene in human) is activated by bile acids to bind to FXR response elements as monomers or as heterodimers with RXR and regulate the expression of diverse genes involved in the metabolism of bile acids, lipids, and carbohydrates [53]. So far, few studies have focused on the molecular effects of FXR activation in prostate cancer. FXR expression was significantly lower in prostate cancer tissues compared with nonmalignant tissues [54]. In addition, FXR and its agonists inhibited cell proliferation in LNCaP and PC-3 cells, suggesting FXR as a potential prevention and therapeutic target for prostate cancer [54,55,56].

#### 2.2.5. Liver X Receptors (LXRs)

The LXR subfamily (LXRα and LXRβ) oxysterol-activated receptors bind to LXR responsive elements as heterodimers with RXRs and regulate the expression of target genes that are involved in lipid metabolism as sensors of cholesterol homeostasis [57]. LXRs are expressed in epithelial and stromal cells of prostate [58]. However, the expression of LXRs was decreased during the progression from hormone-naïve to castration-resistant tumors in a xenograft model [59]. Although the effect of LXR agonists on AR signaling is controversial, knockdown of LXRα and LXRβ revealed no direct effect of LXRs on AR signaling (Figure 1) [60,61]. LXR-deficient mice fed a high cholesterol diet presented prostatic intraepithelial neoplasia (PIN) [62]. In addition, activation of LXRs by their agonists decreased prostate cancer cell proliferation and prostate tumor invasion [59,63,64,65,66,67]. Thus, these data indicate that LXRs play an antioncogenic role in prostate cancer, suggesting that selective LXR modulators could be novel prevention and therapeutic strategies for prostate cancer [68].

#### 2.2.6. Vitamin D Receptor (VDR)

VDR is activated by vitamin D (1,25-(OH)_2_D_3_) and acts as a transcription factor by heterodimerizing with RXR, migrating into the nucleus, and binding to vitamin D responsive elements [69]. VDR has been extensively investigated in association with prostate cancer pathogenesis. High VDR expression in prostate cancer clinical samples was associated with a reduced risk of lethal cancer, suggesting an antioncogenic role of the vitamin D pathway in prostate cancer progression [70]. Numerous epidemiological studies have shown the association between the amount of dietary vitamin D, vitamin D level in circulation, and sunlight exposure with prostate cancer risk [71]. In addition, genetic polymorphisms in VDR were associated with prostate cancer susceptibility and prostate cancer progression [72,73,74]. VDR-knockout mice showed higher cell proliferation than wild-type mice [75]. Consistent with these findings, numerous studies have demonstrated the anticancer effects of vitamin D treatment [76]. Based on these preclinical studies, vitamin D compounds such as calcitriol were examined as single agents as well as in combination with cytotoxic agents such as docetaxel in clinical trials and showed some clinical response such as slowing PSA elevation [76]. The phase III ASCENT I trial was promising, which showed possible favorable antitumor effects such as PSA decline and survival by calcitriol with weekly docetaxel [77]. However, the subsequent phase III ASCENT II trial was disappointing, showing detrimental survival by calcitriol with 3-weekly docetaxel [78].

### 2.3. Subfamily 2

This subfamily consists of hepatocyte nuclear factor-4, RXRs, testicular orphan nuclear receptors (TRs), and COUP-TFs [3].

#### 2.3.1. Retinoid X Receptors (RXRs)

The RXR subfamily (RXRα, RXRβ, and RXRγ) plays diverse roles as NRs because they function either as homodimers that bind to direct repeat sites in gene promoters or as heterodimers with other receptors such as PPARs, LXRs, or FXRs [79]. RXRs are activated by the potent natural ligand 9-cis-retinoic acid as well as all-trans-retinoic acid and novel ligands such as fatty acid and phytanic acid [80]. Previous studies showed that the expression of RXRα was reduced in prostate cancer compared with nonmalignant prostate [35,81,82]. Inactivation of RXRα in the prostate epithelium led to the development of preneoplastic lesions in mice [83], while RXRα overexpression caused cell growth reduction or increased susceptibility to apoptosis in prostate cancer cells [81]. Consistent with these findings, RXR agonists suppressed cell growth and multidrug resistance in prostate cancer both alone [26,84] and in combination with insulin-like growth factor binding protein-3 [85]. These data suggest that RXRs play an antioncogenic role in prostate cancer with or without interactions with other NRs.

#### 2.3.2. Testicular Orphan Nuclear Receptors (TRs)

TRs are classified as orphan receptors but can be activated by several natural molecules, their metabolites, and synthetic compounds including polyunsaturated fatty acids and the metabolites 13-hydroxyoctadecadienoic acid and 15-hydroxyeicosatetraenoic acid, as well as the antidiabetic drug thiazolidinedione [86]. Two TRs, TR2 and TR4, control the expression of target genes and play several roles in physiological and pathological conditions [86]. Only few studies have reported on the molecular effects of TRs in prostate cancer. TR4-knockout mice developed PIN and prostate cancer, indicating that the TR4 gene is a tumor suppressor gene in prostate carcinogenesis [87]. In contrast, TR4 also plays essential functions in various processes related to prostate cancer progression such as invasion and migration as well as cellular resistance to chemotherapy and radiotherapy [88,89,90,91,92,93]. Taken together, these findings suggest complex functions of TRs in prostate cancer biology.

#### 2.3.3. Chicken Ovalbumin Upstream Promoter-Transcription Factors (COUP-TFs)

Orphan receptor COUP-TFs repress gene expression by directly binding to direct repeat sites in genes and they also activate gene expression through forming a modulatory complex with the Sp1 transcription factor [94]. Only few studies have reported on the molecular effects of COUP-TFs in prostate cancer. Genome-wide analysis on the target genes of AR indicated a direct negative regulation of COUP-TFα by AR, in which COUP-TFα localized in the nucleus in prostate cancer epithelium but not in nonmalignant prostate epithelium [95]. COUP-TFβ also functioned as a corepressor of AR and inhibited androgen-dependent proliferation in LNCaP cells [96]. In contrast, another study indicated that COUP-TFβ expression restored cell proliferation and migration inhibited by miR-382 in PC-3, DU-145, LNCaP, and castration-resistant 22Rv1 cells [97]. Taken together, these findings suggest complex roles of COUP-TFs in prostate cancer biology.

### 2.4. Subfamily 3

This subfamily consists of the steroid receptors (SRs) including estrogen receptors (ERs), estrogen-related receptors (ERRs), AR, glucocorticoid receptor (GR), mineralocorticoid receptor (MR), and progesterone receptor (PR) [3]. SRs are activated by cholesterol-derived hormones and translocate into the nucleus to modulate their target gene expressions, which lead to regulation of various processes in development, metabolism, and reproduction [3]. Cholesterol-derived hormones regulate SRs through direct binding [3]. AR is a leading player in prostate cancer pathogenesis and exerts its functional effect mainly through genomic pathway (Figure 1) [1]. Numerous excellent reviews have already summarized the roles of AR [98,99] and therefore we have omitted a summary on AR in this review.

#### 2.4.1. Estrogen Receptors (ERs)

The ER subfamily proteins (ERα and ERβ) are activated upon binding with estrogen, form a dimer and bind to estrogen response elements in the genome to regulate the expressions of target genes [100]. ERα is highly expressed in female reproductive organs, whereas ERβ is abundantly expressed in the prostate, bladder, lung, testis, brain, and bone [100]. Previous studies showed that ERα was upregulated during malignant transformation of the prostatic epithelium and in high-grade and metastatic prostate cancer as well as CRPC, in which ERα expression was increased by androgen-deprivation therapy, implicating an oncogenic role of ERα [101,102,103]. Consistent with these findings, ERα-knockout mice showed no development of high-grade PIN or prostate cancer in experimental carcinogenesis by chronic treatment with testosterone and estradiol [104]. Based on these findings, ERα inhibitors such as the antiestrogen fulvestrant and ERα antagonist toremifene were examined in early-stage clinical trials and showed potential antitumor activity in prostate cancer [105,106].

In contrast to ERα, ERβ preferentially binding to phytoestrogens is likely to protect the prostate epithelium from malignant transformation [103]. ERβ was expressed at high levels in luminal cells of the prostatic epithelium but was partly lost in high-grade PIN, suggesting that ERβ acts as a tumor suppressor [107]. In addition, ERβ expression was suppressed by androgen-deprivation therapy [108]. In studies using ERβ-knockout mice, ERβ was shown to downregulate AR signaling via inducing the AR corepressor dachshund family in the prostate [109]. Consistent with these findings, ERβ agonist was shown to suppress AR expression, resulting in decreased cell survival and increased apoptosis in androgen-dependent VCaP cells [110]. Taken together, these results suggest that ERs may be novel prevention and therapeutic targets for prostate cancer.

#### 2.4.2. Estrogen-Related Receptors (ERRs)

The ERRs (ERRα, ERRβ, and ERRγ) share a high degree of homology with ERs within the DNA-binding domain and LBD, but ERRs do not bind to estrogen [111]. ERRα was expressed in prostate cancer [112] and its expression further increased in aggressive disease [113,114]. In addition, ERRα expression was higher in bone metastases in CRPC than in primary hormone-naïve prostate cancer [115]. These findings suggest that ERRα might play an oncogenic role in the development and progression of prostate cancer. In contrast, ERRβ and ERRγ expressions were decreased in prostate cancer and further decreased in aggressive disease [112,114,116]. Consistent with these findings, overexpression of ERRβ or ERRγ suppressed cell proliferation in prostate cancer cells, suggesting that these receptors exhibit antioncogenic functions in prostate cancer [117,118].

#### 2.4.3. Glucocorticoid Receptor (GR)

Various subtypes of GR are derived from a single gene by alternative splicing and alternative translation initiation mechanisms [119]. Upon binding to glucocorticoids, GR translocates to the nucleus as a homodimer, binds to glucocorticoid response elements in the promoter of target genes, and regulates the expression of target genes that function in broad physiological and pathological processes such as cell growth, energy production, metabolic processes, reproduction, and immune and cardiovascular function [120]. GR expression was reduced in prostatic cancer cells to lower levels than in epithelium of BPH [121] while GR expression was increased in malignant-transformed prostate epithelial RWPE-2 cells [7], indicating controversies on GR expression in prostate cancer. Classically, glucocorticoids were shown to exert antitumor effects in prostate cancer, in which glucocorticoid suppressed adrenal androgens in men with prostate cancer [119]. In addition, the potential for glucocorticoids to promote rather than to suppress prostate cancer growth has been raised based on the structural similarity to AR [122]. In GR-overexpressing cells, glucocorticoids have been shown to activate a transcriptional program that overlaps with genes induced by AR activation (Figure 1) [123]. Therefore, GR may maintain AR signaling in androgen-deprived environments by hijacking the transcriptional program of AR in prostate cancer cells. Previous studies showed that GR expression can be negatively regulated by AR signaling and increased after castration [119,124]. Consistent with these results, some reports demonstrated that GR plays an important role in resistance to androgen-deprivation therapy [125] and the antiandrogen enzalutamide [126]. Taken together, these findings suggest complex roles of GR in prostate cancer biology.

#### 2.4.4. Mineralocorticoid Receptor (MR)

MR is activated primarily by mineralocorticoid aldosterone, but also by glucocorticoids, and translocates to the nucleus as a homodimer where it binds to mineralocorticoid response elements in gene promoters to regulate the expression of genes involved in physiological and pathological conditions in kidney and cardiovascular systems [127]. MR is expressed in both LNCaP and PC-3 cells [128]. Mineralocorticoids such as corticosterone and deoxycorticosterone have been shown to inhibit AR activity in the presence of androgens [129]. In addition, MR suppression by siRNA or antagonists such as spironolactone and eplerenone have been shown to activate AR signaling, suggesting an antagonistic activity to AR [130,131]. Consistent with these findings, mineralocorticoids such as corticosterone and deoxycorticosterone inhibited cell proliferation in androgen-dependent LAPC-4 and LNCaP cells in the presence of androgens [129], while MR antagonists increased LNCaP cell viability [132]. Moreover, MR signaling augmented cellular sensitivity to the antiandrogen enzalutamide [131]. Inversely, the MR antagonist was suggested to promote resistance to AR-targeting therapies [133]. Intriguingly, a genetic polymorphism in MR was associated with prognosis in androgen-deprivation therapy for metastatic prostate cancer, suggesting that MR plays a key role in the resistance of prostate cancer to AR axis-targeting therapies [134].

#### 2.4.5. Progesterone Receptor (PR)

Among the PR isoforms (PRA, PRB, and PRC), PRA and PRB are derived from a single gene by alternative transcription initiation and these proteins represent the major functional isoforms [135]. Upon binding to progesterone, PR translocates to the nucleus as a homodimer, binds to progesterone response elements in the promoter of target genes, and regulates the expression of genes involved in developmental processes as well as proliferation and differentiation during the reproductive cycle and pregnancy in female reproductive tissues [136]. PR was expressed in prostate stroma, while PR expression in the prostate epithelium is controversial [137]. However, PR expression was reduced in cancer-associated stroma [138]. Consistent with these findings, stromal PR suppressed cancer cell migration and invasion via a paracrine mechanism of the stromal cell derived factor-1 and interleukin-6 [139], suggesting an antioncogenic function of PR in stromal cells. Furthermore, the expression level of PR in cancer cells increased with Gleason score, tumor progression, and clinical failure [140,141,142]. Interestingly, PR expression was increased after androgen-deprivation therapy, suggesting a negative regulation of PR expression by AR [137]. These results suggest that PR in cancer cells and the associated stromal cells distinctly regulate prostate cancer pathogenesis.

### 2.5. Subfamilies 5 and 6

Subfamily 5 contains steroidogenic factor-1 (SF-1) and liver receptor homolog-1 (LRH-1), which are generally still classified as orphan receptors, but phospholipids were suggested as possible ligands. These NRs function as a monomer and are required for development and metabolism [3]. Subfamily 6 contains only one orphan receptor, germ cell nuclear factor (GCNF), which is critical for development. The few available studies on these receptors have indicated a role in prostate cancer pathogenesis [3].

SF-1, a key regulator of steroidogenesis in normal endocrine tissues, is not expressed in benign cells, but present in prostate cancer cell lines [143]. SF-1 overexpression in benign prostate cells stimulated steroidogenic enzyme expression, steroid synthesis, and cell proliferation. Furthermore, SF-1 was required for steroid-mediated cell growth in prostate cancer cells [143]. Similarly, increased expression of LRH-1 was detected in high-grade prostate cancer and CRPC xenograft models [144]. LRH-1 was shown to promote de novo androgen biosynthesis via direct transactivation of several key steroidogenic enzyme genes, resulting in elevation of intratumoral androgen levels and reactivation of AR signaling in CRPC xenografts as well as abiraterone-treated CRPC tumors [144]. Thus, these NRs may promote prostate cancer progression via regulating steroidogenesis (Figure 1). Cellular levels of GCNF expression were higher in prostate cancer compared with normal prostate and further increased in metastatic lesions and CRPC [145], suggesting an oncogenic role of GCNF via an unknown mechanism.

## 3. Conclusions and Future Directions

The current literature on the functions of NRs in prostate cancer indicate that DAX1, SHP, RARβ/γ, FXRs, LXRs, VDR, ERβ, ERRβ/γ, and MR can play antioncogenic roles, while PPARγ, RORγ, SF-1, LRH-1, Erα, and GR, as well as AR, can play oncogenic roles (Table 1). However, the role of other NRs in prostate cancer remains controversial or uninvestigated, suggesting a need for further research. In particular, extensive research focusing on SRs in subfamily 3 including AR and other AR-mimic SRs should be required as these NRs are considered to play critical roles in prostate cancer pathogenesis. In addition, based on the recent demonstrated findings from other NRs in prostate cancer, examination of these NRs may lead to the development of novel preventions and therapeutics for prostate cancer.

## Figures and Tables

**Table 1:** Nuclear receptor superfamily proteins and their roles in prostate cancer.

Family	Common Name	Abbreviation	GENE Name	Ligand	Gene Expression in Prostate Tissues (Ref. [7])	Expression in Prostate Cancer	Effect on AR Signaling	Oncogenic Role in Prostate Cancer	References
0B	Dosage-sensitive sex reversal-adrenal hypoplasia congenital critical region on the X chromosome, Gene 1	DAX1	*NR0B1*	Orphan	Detectable	Controversial (increased/decreased)	Suppressive	-	[9,10,11,12]
	Short heterodimeric partner	SHP	*NR0B2*	Orphan	Non-detectable	Decreased	Suppressive	Suppressive	[14,15,16,17,18,19]
1A	Thyroid hormone receptor-α	TRα	*THRA*	Thyroid hormones	Detectable	-	-	-	-
	Thyroid hormone receptor-β	TRβ	*THRB*	Thyroid hormones	Non-detectable	-	-	-	-
1B	Retinoic acid receptor-α	RARα	*RARA*	Retinoic acids	Non-detectable	-	-	-	-
	Retinoic acid receptor-β	RARβ	*RARB*	Retinoic acids	Detectable	Decreased	-	Suppressive	[23,24,26,27,28,29,31]
	Retinoic acid receptor-γ	RARγ	*RARG*	Retinoic acids	Non-detectable	Decreased	Suppressive	Suppressive	[7,21,22,24,25,26,27,28,29,30]
1C	Peroxisome proliferator-activated receptor-α	PPARα	*PPARA*	Fatty acids	Detectable	No change	-	-	[34]
	Peroxisome proliferator-activated receptor-β	PPARβ	*PPARD*	Fatty acids	Detectable	No change	-	Controversial (promotive/suppressive)	[34,44,45,46]
	Peroxisome proliferator-activated receptor-γ	PPARγ	*PPARG*	Fatty acids	Non-detectable	Increased	Controversial(promotive/suppressive)	Promotive	[34,35,36,37,38,39,40,41,42,43]
1D	Reverse-Erb-α	REV-ERBα	*NR1D1*	Heme	Non-detectable	-	-	-	-
	Reverse-Erb-β	REV-ERBβ	*NR1D2*	Heme	Detectable	-	-	-	-
1F	Retinoic acid receptor-related orphan receptor-α	RORα	*RORA*	Sterols	Detectable	Decreased		Suppressive	[51,52]
	Retinoic acid receptor-related orphan receptor-β	RORβ	*RORB*	Sterols	Detectable	-	-	-	-
	Retinoic acid receptor-related orphan receptor-γ	RORγ	*RORC*	Sterols	Non-detectable	Increased	Promotive	Promotive	[49,50]
1H	Farnesoid X receptor-α	FXRα	*NR1H4*	Bile Acids	Detectable	Decreased	-	Suppressive	[54,55,56]
	Farnesoid X receptor-β	FXRβ	*NR1H5P*	Orphan	-	Decreased	-	Suppressive	[54,55,56]
	Liver X receptor-α	LXRα	*NR1H3*	Oxysterols	Detectable	Decreased	None	Suppressive	[7,58,59,60,61,62,63,64,65,66,67]
	Liver X receptor-β	LXRβ	*NR1H2*	Oxysterols	Detectable	Decreased	None	Suppressive	[7,58,59,60,61,62,63,64,65,66,67]
1I	Vitamin D receptor	VDR	*VDR*	1α,25-dihydroxyvitamin D3	Non-detectable	-	-	Suppressive	[70,71,72,73,74,75]
	Pregnane X receptor	PXR	*NR1I2*	Endobiotics and xenobiotics	Non-detectable	-	-	-	-
	Constitutive androstane receptor	CAR	*NR1I3*	Xenobiotics	Non-detectable	-	-	-	-
2A	Hepatocyte nuclear factor-4-α	HNF4α	*HNF4A*	Fatty acids	Detectable	-	-	-	-
	Hepatocyte nuclear factor-4-γ	HNF4γ	*HNF4G*	Fatty acids	Non-detectable	-	-	-	-
2B	Retinoid X receptor-α	RXRα	*RXRA*	9-cis retinoic acid	Detectable	Decreased	-	Suppressive	[7,26,35,81,82,83,84]
	Retinoid X receptor-β	RXRβ	*RXRB*	9-cis retinoic acid	Detectable	-	-	Suppressive	[26,84,85]
	Retinoid X receptor-γ	RXRγ	*RXRG*	9-cis retinoic acid	Detectable	-	-	-	-
2C	Testicular orphan nuclear receptor 2	TR2	*NR2C1*	Orphan	Non-detectable	-	-	-	-
	Testicular orphan nuclear receptor 4	TR4	*NR2C2*	Orphan	Detectable	-	-	Controversial (promotive/suppressive)	[87,88,89,90,91,92,93]
2E	Tailless homolog orphan receptor	TLX	*NR2E1*	Orphan	Non-detectable	-	-	-	-
	Photoreceptor-cell-specific nuclear receptor	PNR	*NR2E3*	Orphan	Non-detectable	-	-	-	-
2F	Chicken ovalbumin upstream promoter-transcription factor α	COUP-TFα	*NR2F1*	Orphan	Detectable	Increased	-	-	[95]
	Chicken ovalbumin upstream promoter-transcription factor β	COUP-TFβ	*NR2F2*	Orphan	Detectable	-	Suppressive	Controversial (promotive/suppressive)	[96,97]
	Chicken ovalbumin upstream promoter-transcription factor γ	COUP-TFγ	*NR2F6*	Orphan	Detectable	-	-	-	-
3A	Estrogen receptor-α	ERα	*ESR1*	Estrogens	Detectable	Increased	-	Promotive	[101,102,103,104]
	Estrogen receptor-β	ERβ	*ESR2*	Estrogens	Detectable	Decreased	Suppressive	Suppressive	[103,107,108,109,110]
3B	Estrogen-related receptor-α	ERRα	*ESRRA*	Orphan	Detectable	Increased	-	-	[112,113,114]
	Estrogen-related receptor-β	ERRβ	*ESRRB*	Orphan	Non-detectable	Decreased	-	Suppressive	[112,114,116,117,118]
	Estrogen-related receptor-γ	ERRγ	*ESRRG*	Orphan	Non-detectable	Decreased	-	Suppressive	[112,114,116,117,118]
3C	Androgen receptor	AR	*AR*	Androgens	Non-detectable	Increased	Promotive	Promotive	[98,99]
	Glucocorticoid receptor	GR	*NR3C1*	Glucocorticoids	Detectable	Controversial (increased/decreased)	Promotive	Promotive	[7,119,121,122,123,124,125,126]
	Mineralocorticoid receptor	MR	*NR3C2*	Mineralocorticoids and glucocorticoids	Non-detectable	-	Suppressive	Suppressive	[128,129,130,131,132,133,134]
	Progesterone receptor	PR	*PGR*	Progesterone	Non-detectable	Controversial (increased/decreased)	Unknown	Suppressive	[136,137,138,139,140,141,142]
4A	Nerve growth factor 1B	NGF1-B	*NR4A1*	Orphan	Detectable	-	-	-	-
	Nurr-related factor 1	NURR1	*NR4A2*	Unsaturated fatty acids	Non-detectable	-	-	-	-
	Neuron-derived orphan receptor-1	NOR-1	*NR4A3*	Orphan	Non-detectable	-	-	-	-
5A	Steroidogenic factor-1	SF-1	*NR5A1*	Phospholipids	Detectable	Increased	Promotive	Promotive	[143]
	Liver receptor homolog-1	LRH-1	*NR5A2*	Phospholipids	Detectable	-	Promotive	Promotive	[144]
6A	Germ cell nuclear factor	GCNF	*NR6A1*	Orphan	Non-detectable	Increased	-	-	-

-, Not investigated.

## Abbreviations

AR	Androgen receptor
BPH	Benign prostatic hyperplasia
COUP-TF	Chicken ovalbumin upstream promoter transcription factor
CRPC	Castration-resistant prostate cancer
DAX1	Dosage-sensitive sex reversal-adrenal hypoplasia congenital critical region on the X chromosome, Gene 1
ER	Estrogen receptor
ERR	Estrogen-related receptor
FXR	Farnesoid X receptor
GCNF	Germ cell nuclear factor
GR	Glucocorticoid receptor
LRH-1	Liver receptor homolog-1
LXR	Liver X receptor
MR	Mineralocorticoid receptor
NR	Nuclear receptor
PIN	Prostatic intraepithelial neoplasia
PPAR	Peroxisome proliferator activated receptor
PR	Progesterone receptor
RAR	Retinoic acid receptor
REV-ERB	Reverse-Erb receptor
ROR	Retinoic acid receptor-related orphan receptor
RXR	Retinoid X receptor
SF-1	Steroidogenic factor-1
SHP	Small heterodimer partner
SR	Steroid receptor
TR	Testicular orphan nuclear receptor
VDR	Vitamin D receptor
